# Etching of Uncompensated Convex Corners with Sides along <n10> and <100> in 25 wt% TMAH at 80 °C

**DOI:** 10.3390/mi11030253

**Published:** 2020-02-27

**Authors:** Milče M. Smiljanić, Žarko Lazić, Vesna Jović, Branislav Radjenović, Marija Radmilović-Radjenović

**Affiliations:** 1 Institute of Chemistry, Technology and Metallurgy-Centre for Microelectronic Technologies (IHTM-CMT), University of Belgrade, Njegoševa 12, 11000 Belgrade, Serbia; zlazic@nanosys.ihtm.bg.ac.rs (Ž.L.); vjovic@nanosys.ihtm.bg.ac.rs (V.J.); 2 Institute of Physics, University of Belgrade, Pregrevica 118, 11080 Belgrade, Serbia; bradjeno@ipb.ac.rs (B.R.); marija@ipb.ac.rs (M.R.-R.)

**Keywords:** no convex corner compensation, parallelogram, silicon, wet etching, tetramethylammonium hydroxide (TMAH)

## Abstract

This paper presents etching of convex corners with sides along <n10> and <100> crystallographic directions in a 25 wt% tetramethylammonium hydroxide (TMAH) water solution at 80 °C. We analyzed parallelograms as the mask patterns for anisotropic wet etching of Si (100). The sides of the parallelograms were designed along <n10> and <100> crystallographic directions (1 < n < 8). The acute corners of islands in the masking layer formed by <n10> and <100> crystallographic directions were smaller than 45°. All the crystallographic planes that appeared during etching in the experiment were determined. We found that the obtained types of 3D silicon shape sustain when n > 2. The convex corners were not distorted during etching. Therefore, no convex corner compensation is necessary. We fabricated three matrices of parallelograms with sides along crystallographic directions <310> and <100> as examples for possible applications. Additionally, the etching of matrices was simulated by the level set method. We obtained a good agreement between experiments and simulations.

## 1. Introduction

Anisotropic wet etching of a (100) silicon substrate in 25 wt% tetramethylammonium hydroxide (TMAH) water solution was intensively studied [1,2,3,4,5,6,7,8,9,10,11,12,13,14,15,16,17,18,19,20,21,22,23,24,25,26,27,28,29]. Etched silicon shapes are limited by mask pattern designs and the etching anisotropy of TMAH water solution. Convex corners of the island patterns in the masking layer can be distorted during etching. The fabrication of complex 3D silicon structures requires knowledge of the mechanisms behind the evolution of convex corner compensation during etching [14,15,16,17,18,19,20,21,22,23,24]. Appropriate convex corner compensation depends on the design of the 3D silicon structure. Some compensation techniques can leave not-so-negligible remnants at the bottom of the structure. These remnants can affect the performance of microdevices made using silicon wet etching.

In previous studies, silicon wet etching [1,2,3,4,5,6,7,8,9,10,11,12,13,14,15,16,17,18,19,20,21,22,23,24,25,26,27,28,29] has been conducted using various etching solutions of TMAH at different temperatures, and silicon wafers of various crystallographic orientations. Most of the results were obtained for the etching of square or rectangular patterns in the masking layer with sides along <110> crystallographic directions. Additionally, convex corner compensation techniques for TMAH water solution etching have been developed for patterns with sides along <110> crystallographic directions [14,15,16,17,18,19,20,21,22,23,24]. The etching of a (100) silicon substrate in TMAH water solution using square, rectangular and octagonal mask patterns with sides along different crystallographic directions was explored in [9,27]. The etching of square patterns with sides along <100> crystallographic directions was analyzed in [9,27]. In our previous study [27], we provided a comprehensive study of the etching of square patterns with sides along <n10> crystallographic directions. The etching of octagonal patterns with sides along <210>, <310> and <410> crystallographic directions was discussed in [9] for TMAH water solutions. In [28], authors explored the etching of (110) silicon using parallelograms with sides along <110> and <211> crystallographic directions.

This paper presents our further work on (100) silicon etching in 25 wt% TMAH water solution at 80 °C. We analyzed silicon etching of masks in the shape of parallelograms with sides along determined crystallographic directions <n10> (1 < n < 8) and <100>. The parallelograms were designed as islands of silicon dioxide. We showed that etching of these patterns enables the fabrication of sustainable types of 3D silicon shape. We also observed that convex corners are stable during etching. Such etching is, in our view, very practical, as it does not require compensation for convex corners.

Methods for simulating the etching process fall into two categories. The first category describes the etching process on the atomistic level, usually including a description of the etched surface morphology. The so-called atomistic simulators, based on cellular automata and kinetic Monte Carlo methods [1,30], belong to this group. In these methods, a silicon substrate is represented by a large number of cells that reside in a crystalline lattice. During the etching simulation, the state of each individual cell, whether it is removed from or remains within the lattice, is determined by the strength of its chemical bonds and the link status of its lattice neighbors, expressed by the neighborhood-dependent removal rates. Although these methods can satisfy the accuracy and speed requirements, they have many disadvantages. For instance, there are too many parameters in the simulation model (hundreds or even thousands, including removal rates and others), which should be calibrated using experimentally obtained angular dependence of etching velocities. Also, new calibrations are needed each time the experimental conditions are changed, such as the etchant type, concentration and/or temperature, with calibration lasting several hours or even days [31,32,33].

The second category is the so-called geometric method [34], which does not have such problems. The etching profile is viewed as a set of planes propagating along their normal directions with velocities obtained either experimentally or in other calculations. These simulations require knowledge of the complete angular dependence of the etching rates, but they are here used directly, without an intermediate calibration step. Generally, the continuum geometric representation of the etched surfaces is much more convenient for engineering applications. The most significant example of these methods is the level set method introduced by Osher and Sethian [35], which is widely used for analyzing and computing moving fronts in a variety of settings. The level set method for evolving interfaces is specially designed for profiles that can develop sharp corners or undergo a change of topology, or when the normal component of the velocity on the interface points undergoes significant changes in speed. Our three-dimensional (3D) anisotropic etching simulator is based on the sparse field method for solving the level set equations, and it was described in detail in our previous publications [36,37,38,39]. In this paper, we present a comparison of the results of level set simulations and etching experiments using three different matrices of parallelograms that are examples of possible future designs.

Squares (rectangulars), triangles and circles were the basic patterns for a great majority of mask designs. Undistorted convex corners lead toward a novel design method for angle deflection from the standard <100> direction by forcing the sides of mask patterns along predetermined <n10> crystallographic directions. The analyzed parallelograms could be used for future designs of lab-on-chip platforms based on obstacle mechanisms (micromixers, deterministic lateral displacement (DLD) separators, cell peg) [40,41,42,43,44,45,46,47,48,49,50], microfluidic diodes [51] and quality microfluidic bifurcations. This paper will provide guidance on novel micromachining of bosses, convex corner compensation, microfluidic channels with obstacles, etc.

## 2. Experimental Setup

Phosphorus-doped (100) oriented 3” silicon wafers (Wacker, Munich, Germany; SWI, Hsinchu, Taiwan) with mirror-like single or double side polished surfaces and 1–5 Ω·cm resistivity were used. Additionally, silicon-on-insulator (SOI) wafers (MEMS Material & Engineering, INC, Sunnyvale, CA, USA) were used as examples of 3D silicon structures. The active layer was about 2.5 µm thick phosphorus-doped (100) oriented silicon of 1–5 Ω·cm resistivity. The oxide layer was about 1 µm thick. Anisotropic etching was conducted in pure TMAH 25 wt% water solution (Merck, Darmstadt, Germany). The etching temperature was 80 °C. Wafers were standardly cleaned and covered with SiO_2_, thermally grown at 1100 °C in an oxygen ambient saturated with water vapour. SiO_2_ was etched in buffered hydrofluoric acid (BHF) in a photolithographic process in order to define parallelograms along determined crystallographic directions. Again, wafers were subjected to a standard cleaning procedure and were dipped before etching for 30 s in hydrofluoric acid (HF) (10%) in order to remove native SiO_2_. This was followed by rinsing in deionized water. Etching of the whole 3” wafer was carried out in a thermostated glass vessel containing about 0.8 dm^3^ of the solution, with an electronic temperature controller stabilizing the temperature within ±0.5 °C. The vessel was on the top of a hot plate and closed with a teflon lid, which included a water-cooled condenser to minimize evaporation during etching. The wafer was oriented vertically in a teflon basket inside the glass vessel. Throughout the process, the solution was electromagnetically stirred with a velocity of 300 rpm. After reaching the desired depth, the wafer was rinsed in deionized water and dried with nitrogen.

## 3. Simulation Method

Here we used the level set method introduced by Osher and Sethian [35], a powerful technique for analyzing moving fronts in a variety of different settings. A detailed exposition of the theoretical and numerical aspects of the method can be found in books [52,53], and in numerous review articles. The main idea behind the level set method is to represent the surface at a specified time t as the zero level set of a certain function *φ (t, x).* The velocity of a point on the surface with the direction normal to the surface will be denoted by *R (t, x)* (velocity function) and is completely determined by the physics and chemistry of the ongoing processes. The equation describing the time evolution of the unknown function *φ (t, x)* has Hamilton–Jacobi form:

(1) $$\frac{\partial \varphi }{\partial t}+H\left(\nabla \varphi \left(t,x\right)\right)=0$$

where the Hamiltonian is given by:

(2) H=R(t,x)|∇φ(t,x)|

Many approaches for solving level set equations exist that increase accuracy while decreasing computational effort. The most important are the so-called narrow band level set methods, widely used in etching process modeling tools, and the recently developed sparse-field method [54], used widely in the image processing community. If the surface velocity *R (t, x)* does not depend on the level set function *φ (t, x)* itself, the Hamiltonian function defined by relation (2) is usually convex and the spatial derivatives of *φ (t, x)* can be approximated using the Engquist–Osher upwind finite difference scheme. Unfortunately, the non-convex Hamiltonians are typical for simulations of anisotropic wet etching, plasma etching and deposition. The simplest scheme that can be applied in these cases is the Lax–Friedrichs scheme [52,53], and it is used in our simulation package. The Lax–Friedrichs scheme itself is known as a numerically dissipative scheme, where dissipation causes some smearing of sharp geometric elements. The proper amount of dissipation (dissipation parameters) is a still-active research topic. The same method has been used in a recent implementation of the sparse field LS method described in [31,32]. Our approach is based on the ITK (Insight Toolkit) library [55]. ITK is an open source project inclined toward image processing tasks, but its implementation of the sparse field LS method can be used in any sort of ‘moving interface’ problem.

In order to simulate the time evolution of 3D etching profiles, it is essential that the exact etch rates in all directions are known. The etch rates are known for a rather limited number of directions, but they can be used to determine rate value in an arbitrary direction by an interpolation procedure. The problem of etch rate interpolation is equivalent to function interpolation over a sphere in 3D. The etch rate angular dependence model function must interpolate through the given etch rates and directions while maintaining only C0 continuity, since empirical studies have shown cusps in etch rate diagrams. The simulations presented here are performed using our 13-parameters model, described in detail in [38], with the parameters’ values given in [15].

## 4. Results and Discussion

**Figure 1:**
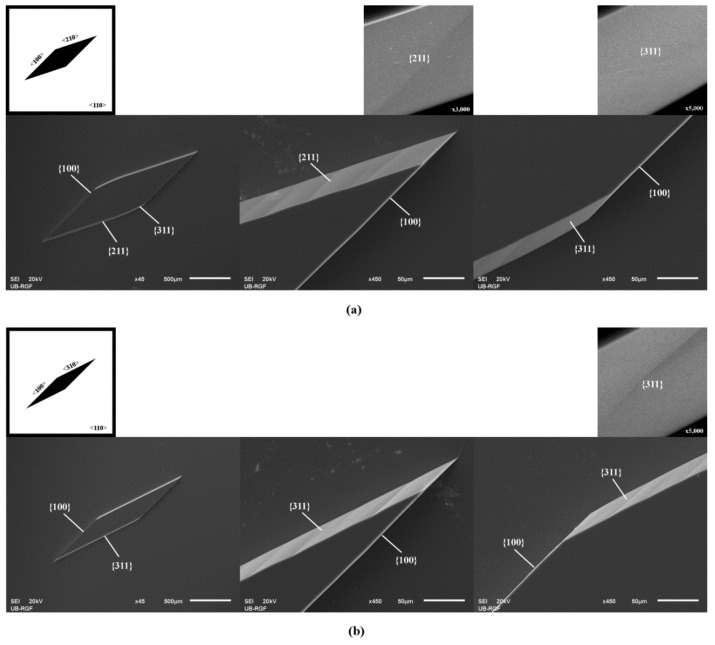
Schematic mask patterns and scanning electron microscope (SEM)micrographs (standard Si wafer) of the etched parallelograms with sides along <100> directions, (**a**) <210> directions and (**b**) <310> directions. Parallelograms are islands of the masking layer (black). Enlarged details of the etched acute and obtuse angles in the masking layer are given for both cases. Magnified {211} and {311} planes are presented.

**Figure 2:**
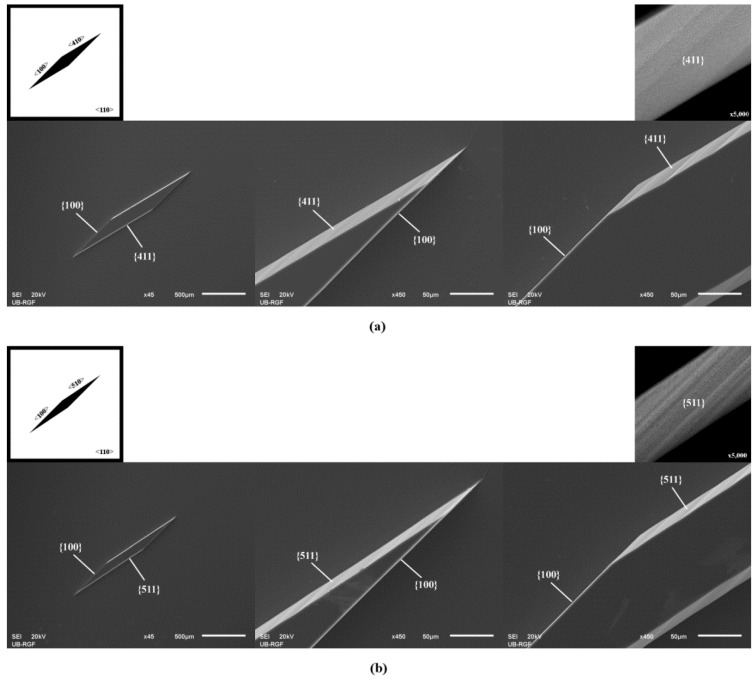
Schematic mask patterns and SEM micrographs (standard Si wafer) of the etched parallelograms with sides along <100> directions, (**a**) <410> directions and (**b**) <510> directions. Parallelograms are islands of the masking layer (black). Enlarged details of the etched acute and obtuse angles in the masking layer are given for both cases. Magnified {411} and {511} planes are presented.

**Figure 3:**
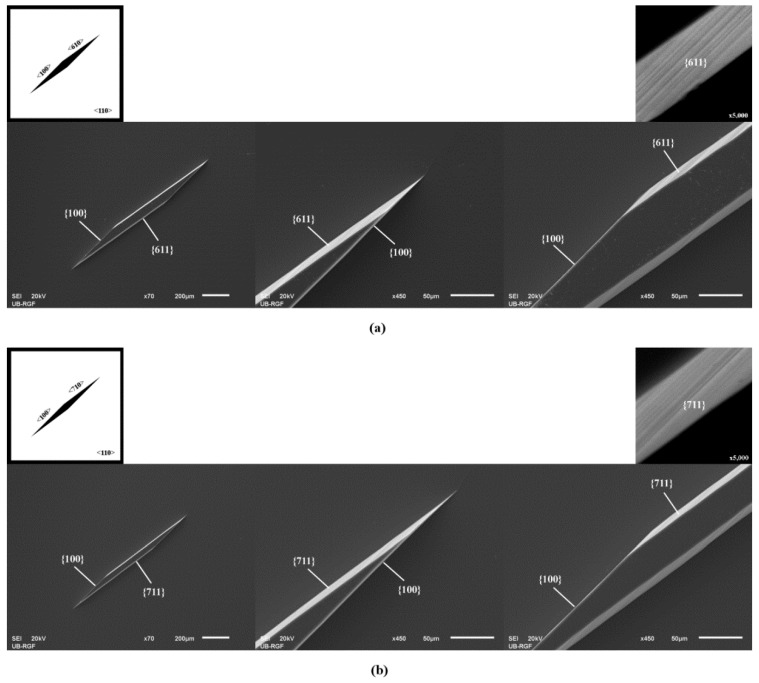
Schematic mask patterns and SEM micrographs (standard Si wafer) of the etched parallelograms with sides along <100> directions, (**a**) <610> directions, and (**b**) <710> directions. Parallelograms are islands of the masking layer (black). Enlarged details of the etched acute and obtuse angles in the masking layer are given for both cases. Magnified {611} and {711} planes are presented.

Parallelograms were designed with sides along determined crystallographic directions <n10> (1 < n < 8) and <100>. The sides of parallelograms were 1500 µm. The acute angles of islands in the masking layer formed by <n10> and <100> crystallographic directions were smaller than 45°. The values of the acute and obtuse angles of the parallelograms are given in Table 1. In our previous work, we presented and analyzed parallelograms with acute angles larger than 45° and smaller than 90° [29]. In this experiment, we etched standard Si (100) wafers. The etch depths in Figure 1, Figure 2 and Figure 3 are 55 µm. The silicon dioxide masking layer was removed after etching. In the first case of n = 2, the obtained 3D silicon shape was a prismoid with parallelograms as its bases, as shown in Figure 1a. The prismoid’s bases were parallel polygons with the same number of sides, and the lateral faces (sidewalls) were all trapezoids or parallelograms [56]. Sidewalls of the prismoid were defined by {100} and {211}–{311} planes. The transition from {211} to {311} was smooth. Along initial <210> crystallographic directions, the sidewalls were defined by {211}–{311} families, as in the case of an etched square with sides along <210> crystallographic directions [27]. At the obtuse angle in the masking layer, the sidewalls were {100} and {311} planes.

In all other cases (n > 2), the sidewalls of prismoids were defined only by {n11} and {100} families during the etching of silicon, as shown in Figure 1b, Figure 2 and Figure 3. The sidewalls of the 3D silicon structure aligned to the <n10> direction belonged to {n11} crystallographic planes, as in [27]. The planes {n11} were inclined at angles with the values given in Table 2. In cases when n > 2, the obtained types of 3D silicon shape sustained during etching. The surfaces corresponding to the sidewalls and bases of consecutively etched 3D shapes were parallel. The corresponding angles did not change during etching. The dimensions of the 3D shape changed over time, as expected. Neither the acute corners nor the obtuse corners in the masking layer required convex corner compensation as they were not distorted during etching. There were no remnants at the bottom of the etched silicon structure, as shown at SEM micrographs in Figure 1, Figure 2 and Figure 3. However, as we already noticed in our previous work [14], there were facets with a weak curvature (FWC) at every joint between the sidewalls and the bottom surface. It was not possible to determine the angle of these facets, as there is a smooth transition from the bottom to the sidewalls.

Convex corner compensations formed as the <100> beams for the etching of square or rectangular patterns with sides along <110> crystallographic directions can be related with parallelograms in the case of n = 3. A pattern formed of two adjoined symmetrical parallelograms, which shared one side along a <100> crystallographic direction, was analyzed. The etched convex corners of the pattern represent the free end of convex corner compensation bounded by {100} and {311} planes [15,16,17,18].

In our previous work [29], we analyzed the evolution of the convex corners of 3D silicon structures etched from parallelograms with acute angles larger than 45° and smaller than 90°. For these types of parallelograms, with sides along <n10> and <100> crystallographic directions, distortion of convex corners appeared.

Figure 1, Figure 2 and Figure 3 show magnified {n11} planes. It can be noticed that planes from the {211} and {311} families are smooth. To be precise, they consist of large smooth surface facets. Planes from the {411}, {511}, {611} and {711} families are not as smooth as planes from the {211} and {311} families. It looks like they consist of dense consecutive facets. The density of facets increases with *n*.

**Figure 4:**
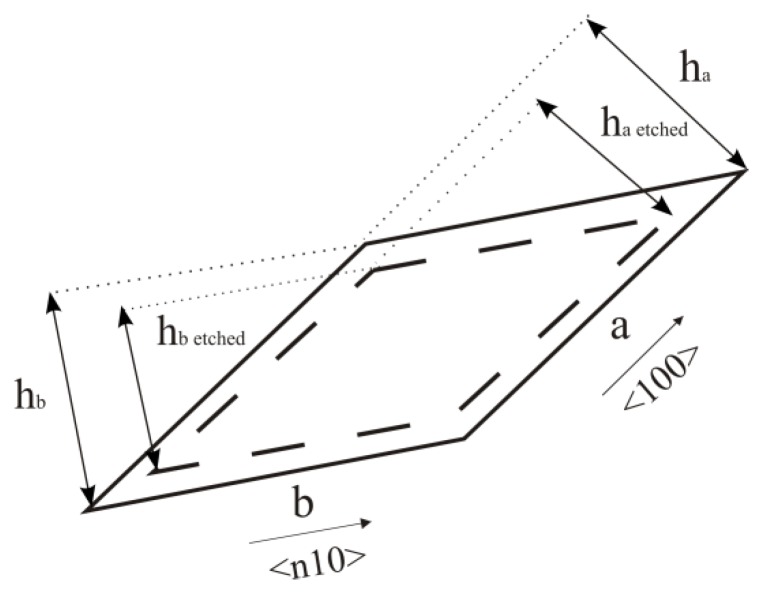
Schematic picture of the parallelogram heights *h_a_* and *h_b_* changing in the masking layer during etching. *a* and *b* are length of the sides of a parallelogram in the <100> and <n10> crystallographic directions respectively.

The heights of the parallelograms in the masking layer, as shown in Figure 4, depend on the etching depth *d*, according to the following equations:

(3) $$h_{a\;\mathrm{etched}}=h_{a}-2d$$

(4) $$\;h_{b\;\mathrm{etched}}=h_{b}-2\frac{r_{n11}}{r_{100}\sin\gamma_{n11}}d=h_{b}-2U_{n11}d\;$$

(5) $$\;U_{n11}=\frac{r_{n11}}{\sin\gamma_{n11}r_{100}}\;$$

Where *h_a_* and *h_a_*_etched_ are the heights of the parallelogram side along the <100> crystallographic direction before and after etching, respectively, *h_b_* and *h_b_*_etched_ are the heights of the parallelogram side along the <n10> crystallographic direction before and after etching, respectively, *r_n_*_11_ and *r*_100_ are the etch rates of the {n11} and {100} crystallographic planes, *γ_n_*_11_ is the angle between the {n11} and {100} crystallographic planes and *U_n_*_11_ is the undercut ratio defined only along one <n10> side of the parallelogram. The values of *r_n_*_11_ and *γ_n_*_11_ are given in Table 2 and Table 3 [27]. The value of *r*_100_ is 0.46 µm/min. Calculated undercut ratios *U_n_*_11_ are given in Table 3. Parameters *2d* and *2U_n_*_11_*d* define the sizes of the smallest structures that would not be undercut during etching.

**Figure 5:**
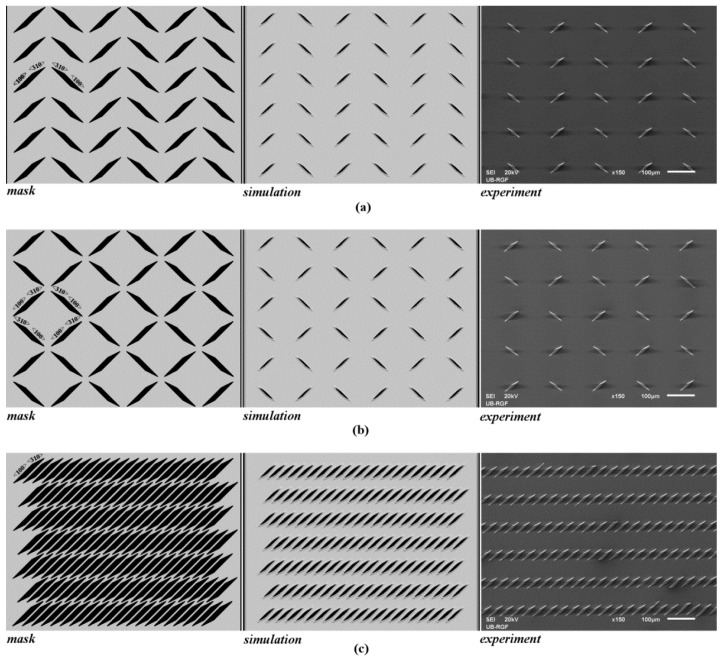
Mask patterns, simulated etching profiles and SEM micrographs (silicon-on-insulator (SOI) active layer) of three matrices of parallelograms with sides along crystallographic directions <310> and <100>: (**a**) fishbone patterns; (**b**) zigzag patterns; and (**c**) directed parallelograms. Etched depths in simulations and experiments are 6 µm.

**Figure 6:**
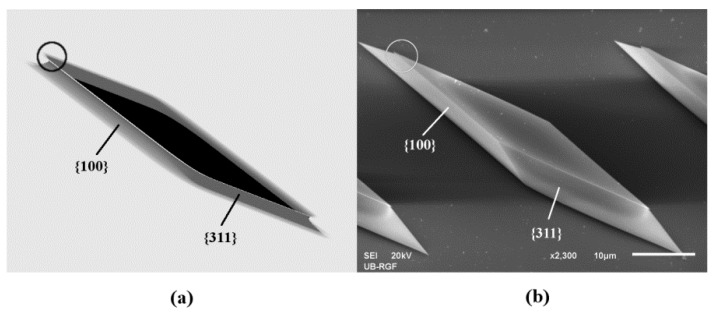
Enlarged 3D silicon shape from Figure 5. The angles of tiltation are 30°. The appearances of the planes directly under the masking layers, which have smaller surfaces than the dominant ones and form shapes resembling ship prows, are marked with circles. (**a**) Simulated etching profile; (**b**) SEM micrograph.

We present etching of the SOI active layer as one example, shown in Figure 5 and Figure 6. Three different matrices of parallelograms with sides along crystallographic directions <310> and <100> were designed, as shown in Figure 5. Before the experiments, we performed 3D simulations of mask patterns during silicon etching based on the level set method. The pictures of the simulated etching profiles were rendered by the Paraview visualization package [57]. Figure 6 shows an enlarged silicon prismoid defined only by the planes of the {311} and {100} families. Appearance of the planes directly under the masking layer can be noticed in Figure 6, which shows the simulated etching profiles [11,15,27]. These planes have smaller surface areas than the dominant ones and form shapes resembling ships’ prows. The planes obtained in simulations are more round and the edges of the convex corners tend to soften. There is a good agreement between experiment and simulation, as shown in Figure 5 and Figure 6. This will allow the use of simulation based on the level set method as a cost-effective tool for future mask designs. The etch depth was 6 µm. The sides of the parallelograms were designed according to Equations (3) and (4), and the symmetry of silicon etching. At the end of etching, heights *h_a_*_etched_ and *h_b_*_etched_ of the parallelogram on the surface of active layer were 10 µm, as designed. The silicon dioxide masking layer was removed after etching.

As convex corner compensation is not necessary, patterns with observed convex corners and parallelograms could be used in future designs of various sensors, actuators and silicon-based platforms. The obtained matrices, shown in Figure 5, can be used as the integrated obstacles in microfluidic channels. Many lab-on-chip platforms are based on obstacle mechanisms like micromixers [40,41,42,43,44,45], deterministic lateral displacement (DLD) separators [46,47,48,49] and cell pegs [50]. Most of these designs use polydimethylsiloxane (PDMS) as the structural material. Micromachining of silicon obstacles using wet chemical etching, together with anodic bonding to Pyrex glass, allows for more rigid platforms [58,59].

Our findings also provide new possibilities for designs that include crystallographic directions different from the standard <100> and <110> directions. This can allow the fabrication of high-quality microfluidic bifurcations, as there are no remnants at the bottom of the etched silicon convex corners. Polygons with sides along the appropriate <n10> and <100> crystallographic directions can also be used for fabrication of microfluidic diodes [51]. Microfluidic diodes based on geometric effects allow flow in a predefined direction and prevent spreading of the same fluid in the opposite direction.

## 5. Conclusions

In this paper, we studied silicon etching of parallelograms as mask patterns using 25 wt% TMAH water solution at 80 °C. Sides of the parallelogram islands were designed along <n10> and <100> crystallographic directions. All crystallographic planes that appeared during etching of silicon structures were determined. We discovered that the types of 3D silicon shape obtained for n > 2 will sustain during etching. The convex corners of the silicon structures are not distorted during etching and no convex corner compensation is needed. Such predictable evolution provides opportunities for new controllable designs of various complex silicon structures that do not use only the most common directions <110> and <100>.

## Figures and Tables

**Table 1:** The values of acute and obtuse angles of the parallelograms.

Crystallographic Direction <n10>	Acute Angle [°]	Obtuse Angle [°]
<210>	26.6	153.4
<310>	18.4	161.6
<410>	14	166
<510>	11.3	168.7
<610>	9.5	170.5

**Table 2:** The values of angles of inclination *γ*_n11_.

Crystallographic Plane {n11}	Angle *γ*_n11_ Theoretical [°]	Angle *γ*_n11_ [27] [°]
<211>	65.9	66.7
<311>	72.5	74.2
<411>	76.4	78.7
<511>	78.9	80.9
<611>	80.7	81

**Table 3:** The values of etch rates and ratios of undercut and etch depth.

Crystallographic plane {n11}	Etch Rates *r_n_*_11_ [27] [µm/min]	*U_n_*_11_
<211>	0.87	2.06
<311>	0.93	2.10
<411>	0.85	1.89
<511>	0.81	1.78
<611>	0.73	1.61
<711>	0.69	1.52
